# Stroke: a global response is needed

**DOI:** 10.2471/BLT.16.181636

**Published:** 2016-09-01

**Authors:** Walter Johnson, Oyere Onuma, Mayowa Owolabi, Sonal Sachdev

**Affiliations:** a Department of Service Delivery and Safety, World Health Organization, avenue Appia 20, 1211 Geneva 27, Switzerland.; b Management of Noncommunicable Diseases, Disability, Violence and Injury Prevention Department, World Health Organization, Geneva, Switzerland.; c Department of Medicine, University of Ibadan, Ibadan, Nigeria.

Worldwide, cerebrovascular accidents (stroke) are the second leading cause of death and the third leading cause of disability.^1^ Stroke, the sudden death of some brain cells due to lack of oxygen when the blood flow to the brain is lost by blockage or rupture of an artery to the brain, is also a leading cause of dementia and depression.^2^ Globally, 70% of strokes and 87% of both stroke-related deaths and disability-adjusted life years occur in low- and middle-income countries.^3^^–^^5^ Over the last four decades, the stroke incidence in low- and middle-income countries has more than doubled. During these decades stroke incidence has declined by 42% in high-income countries.^3^ On average, stroke occurs 15 years earlier in – and causes more deaths of – people living in low- and middle-income countries, when compared to those in high-income countries.^2^ Strokes mainly affect individuals at the peak of their productive life. Despite its enormous impact on countries’ socio-economic development, this growing crisis has received very little attention to date.

The risk factors for stroke are similar to those for coronary heart disease and other vascular diseases. Effective prevention strategies include targeting the key modifiable factors: hypertension, elevated lipids and diabetes. Risks due to lifestyle factors can also be addressed: smoking, low physical activity levels, unhealthy diet and abdominal obesity.^6^ Combinations of such prevention strategies have proved effective in reducing stroke mortality even in some low-income settings.^7^^,^^8^

Furthermore, as most guidelines are based on high-income country data, uncertainty remains regarding best management of stroke of unknown type in low- and middle-income countries. For example, in low- and middle-income countries, 34% of strokes (versus 9% in high-income countries) are of haemorrhagic subtype and up to 84% of stroke patients in low- and middle-income countries (versus 16% in high income countries) die within three years of diagnosis.^2^ Current guidelines for the management of acute stroke recommend a course of treatment based on the diagnosis of ischaemic stroke (versus haemorrhagic stroke) made using computed tomography (CT) scanners. In low-resource settings, CT scanners are either unavailable or unaffordable, forcing clinicians to make difficult clinical decisions, such as whether to anticoagulate patients or not, and to what level to control their blood pressure without a means of distinguishing between ischaemic and haemorrhagic stroke. These patient management challenges, combined with inadequate rehabilitation services, lack of preventive measures, as well as poor understanding of the possible unique risk factors associated with stroke in low- and middle-income countries, may account for the disproportionately large stroke burden borne by these countries.

The reasons for the younger age of onset, higher rates of haemorrhagic subtype and higher case fatality, are unknown.^2^ Better understanding of the possible unique risk factors for this epidemic in low- and middle-income countries is urgently needed. The Stroke Investigative Research and Educational Network study is investigating the underlying risk factors for stroke occurrence, subtype and outcome among people of African ancestry.^9^ Understanding the genetic basis for the interactions between risk factors can inform targeted prevention efforts, as part of a broader approach with four parts: surveillance, prevention, acute care and rehabilitation.^2^ This type of integrated approach will generate the evidence base to produce the guidelines needed for stroke prevention, treatment and rehabilitation in low- and middle-income countries. 

In the July 2016 issue of the *Bulletin*, Aaron Berkowitz^10^ examined current acute stroke management practice in low-resource settings and outlined items for consideration when developing treatment guidelines for patients with acute stroke of unknown etiology in settings where there are no CT scanners. Berkowitz emphasized the proven efficacy of supportive care measures, such as maintaining euglycaemia and euthermia, prevention of deep-vein thrombosis and aspiration, early mobilization and prompt seizure treatment for stoke patients. He recommended judicious use of aspirin and provided blood pressure parameters for stroke patients in these circumstances. He also emphasized the need for secondary prevention. 

Managing acute stroke in low-resource settings requires a novel approach, one that could restart the original WHO global stroke initiative,^11^ as a collaboration between the World Health Organization (WHO), the World Stroke Organization and the World Federation of Neurology, to increase awareness of stroke, generate better surveillance data and guide better prevention and management. The WHO *Package of essential noncommunicable disease interventions for primary health care in low-resource settings* provides protocols for cardiovascular risk reduction and stroke prevention.^12^ WHO will develop guidelines for the management of acute stroke in low- and middle-income countries, and aims to expand training programmes in stroke prevention, treatment and rehabilitation through its partners. 

## References

[1] Global Health Estimates. Geneva: World Health Organization; 2012. Available from: http://www.who.int/healthinfo/global_burden_disease/en/ [cited 2016 June 1].

[2] Owolabi MO, Akarolo-Anthony S, Akinyemi R, Arnett D, Gebregziabher M, Jenkins C, et al.; Members of the H3Africa Consortium. The burden of stroke in Africa: a glance at the present and a glimpse into the future. Cardiovasc J Afr. 2015 Mar-Apr;26(2) Suppl 1:S27–38. 10.5830/CVJA-2015-038 25962945 PMC4557491

[3] Feigin VL, Forouzanfar MH, Krishnamurthi R, Mensah GA, Connor M, Bennett DA, et al.; Global Burden of Diseases, Injuries, and Risk Factors Study 2010 (GBD 2010) and the GBD Stroke Experts Group. Global and regional burden of stroke during 1990-2010: findings from the Global Burden of Disease Study 2010. Lancet. 2014 1 18;383(9913):245–54. 10.1016/S0140-6736(13)61953-4 24449944 PMC4181600

[4] Feigin VL, Lawes CMM, Bennett DA, Barker-Collo SL, Parag V. Worldwide stroke incidence and early case fatality reported in 56 population-based studies: a systematic review. Lancet Neurol. 2009 4;8(4):355–69. 10.1016/S1474-4422(09)70025-0 19233729

[5] Strong K, Mathers C, Bonita R. Preventing stroke: saving lives around the world. Lancet Neurol. 2007 2;6(2):182–7. 10.1016/S1474-4422(07)70031-5 17239805

[6] Johnston SC, Mendis S, Mathers CD. Global variation in stroke burden and mortality: estimates from monitoring, surveillance, and modelling. Lancet Neurol. 2009 4;8(4):345–54. 10.1016/S1474-4422(09)70023-7 19233730

[7] O’Donnell MJ, Xavier D, Liu L, Zhang H, Chin SL, Rao-Melacini P, et al.; INTERSTROKE investigators. Risk factors for ischaemic and intracerebral haemorrhagic stroke in 22 countries (the INTERSTROKE study): a case-control study. Lancet. 2010 7 10;376(9735):112–23. 10.1016/S0140-6736(10)60834-3 20561675

[8] Mayosi BM, Lawn JE, van Niekerk A, Bradshaw D, Abdool Karim SS, Coovadia HM; Lancet South Africa team. Health in South Africa: changes and challenges since 2009. Lancet. 2012 12 8;380(9858):2029–43. 10.1016/S0140-6736(12)61814-5 23201214

[9] Akpalu A, Sarfo FS, Ovbiagele B, Akinyemi R, Gebregziabher M, Obiako R, et al.; SIREN as part of the H3Africa Consortium. Phenotyping Stroke in Sub-Saharan Africa: Stroke Investigative Research and Education Network (SIREN) Phenomics Protocol. Neuroepidemiology. 2015;45(2):73–82. 10.1159/000437372 26304844 PMC4604029

[10] Berkowitz AL. Managing acute stroke in low-resource settings. Bull World Health Organ. 2016 7 1;94(7):554–6. 10.2471/BLT.15.162610 27429496 PMC4933138

[11] Bonita R, Mendis S, Truelsen T, Bogousslavsky J, Toole J, Yatsu F. The global stroke initiative. Lancet Neurol. 2004 7;3(7):391–3. 10.1016/S1474-4422(04)00800-2 15207791

[12] WHO Package of essential noncommunicable (PEN) disease interventions for primary health care in low-resource settings. Geneva: World Health Organization; 2016. Available from: http://www.who.int/nmh/publications/essential_ncd_interventions_lr_settings.pdf[cited 2016 June 28]

